# Evidence for exocellular Arsenic in Fronds of *Pteris vittata*

**DOI:** 10.1038/s41598-017-03194-x

**Published:** 2017-06-06

**Authors:** Rupali Datta, Padmini Das, Ryan Tappero, Pravin Punamiya, Evert Elzinga, Shivendra Sahi, Huan Feng, Jeffrey Kiiskila, Dibyendu Sarkar

**Affiliations:** 1 Department of Biological Sciences, Michigan Technological University, Houghton, MI 49931 USA; 2 Department of Biology, Nazareth College of Rochester, NY, 14618 USA; 3 Photon Sciences Division, Brookhaven National Laboratory, Upton, NY 11973 USA; 4 Parsons, 200 Cottontail Lane South 08873, Somerset, 08873 NJ United States; 5 Department of Earth & Environmental Sciences, Rutgers University, Newark, NJ 07102 USA; 6 Department of Biology, Western Kentucky University, Bowling Green, KY 42101 USA; 7 Department of Earth and Environmental Studies, Montclair State University, Montclair, NJ 07043 USA; 8 Department of Civil, Environmental and Ocean Engineering, Stevens Institute of Technology, Hoboken NJ, 07030 USA

## Abstract

The arsenic (As) hyperaccumulating fern species *Pteris vittata* (PV) is capable of accumulating large quantities of As in its aboveground tissues. Transformation to AsIII and vacuolar sequestration is believed to be the As detoxification mechanism in PV. Here we present evidence for a preponderance of exocellular As in fronds of *Pteris vittata* despite numerous reports of a tolerance mechanism involving intracellular compartmentalization. Results of an extraction experiment show that 43–71% of the As extruded out of the fronds of PV grown in 0.67, 3.3 and 6.7 mM AsV. SEM-EDX analysis showed that As was localized largely on the lower pinna surface, with smaller amounts on the upper surface, as crystalline deposits. X-ray fluorescence imaging of pinna cross-sections revealed preferential localization of As on the pinna surface in the proximity of veins, with the majority localized near the midrib. Majority of the As in the pinnae is contained in the apoplast rather than vacuoles. Our results provide evidence that exocellular sequestration is potentially a mechanism of As detoxification in PV, particularly at higher As concentrations, raising concern about its use for phytoremediation.

## Introduction

The existence of the As hyperaccumulating fern species, *Pteris vittata* (PV) was first reported in 2001^1^. *Pteris vittata* has the ability to uptake and translocate large amounts of As to its shoots^2, 3^. Due to its potential in phytoremediation of As, the mechanism of As hyperaccumulation in PV has been the subject of intense research.

Once As is taken up by roots, translocation to fronds occurs rapidly^4^. AsV is reduced to AsIII by arsenate reductase (AR) in the plant^5^. Earlier reports showed that AsV is reduced to AsIII in the fronds^6^, but later reports suggested that the reduction occurred in the roots^7, 8^. Recently, two constitutively expressed AR proteins in both fronds and roots has been reported in sporophytes of PV^9^. Speciation of As is now believed to be dependent on As concentration in the growth media^10–12^. When AsV levels are low, the reduction can occur in the roots, but at higher concentrations, a part of the reduction occurs in the fronds^12^.

In the fronds, As is concentrated at the tips of the apical pinnae, with levels decreasing toward the basal pinnae^13^. Within the pinnae, AsIII is mostly localized at the tips, in the blades proximal to the veins, and within the pseudoindusium^13^; whereas AsV is mainly localized within the veins. At the cellular level, As was reported to be localized in the upper and lower epidermal cells and trichomes of the fronds^14, 15^. Although several reports have mentioned that As is sequestered in the vacuoles in PV^6, 15^, there seems to be no definitive evidence of the same. One study speculated that As was localized in the vacuoles because SEM-EDXA analysis showed As mainly in the central part of the cells in fresh fronds^6^, and central part of the cells is occupied by vacuoles. In a more recent subcellular fractionation study, 78% of the As in PV pinnae was recovered in the cytoplasmic supernatant fraction^16^ which consists of cell and vacuolar sap, hence the authors speculated that As was localized in vacuoles. Other indirect evidence of vacuolar sequestration originated from the identification of arsenite transporter proteins in PV, localized in vacuolar membranes^17^.

Another strategy adopted by plants to cope with As is to efflux it from roots^18^, which is common in excluder, rather than hyperaccumulator plants^19^. Between 6–20% As is effluxed into the medium by PV roots^20, 21^, compared to 80–90% for rice^22, 23^. No evidence of efflux through PV fronds existed until As leaching from foliage was reported recently using a simulated precipitation experiment^24^, where 2–7% of the total foliar As was leached. Water-soluble As from the apoplast was the primary source of the leached As^24^. Another paper reported the secretions from hydathodes in PV contain high levels of As, using SEM-EDX analysis^25^. To verify and further elucidate the phenomenon of As loss through the aboveground parts of PV, we performed detailed SEM-EDX, X-ray microprobe synchrotron analysis, and XRF mapping of cross sections of pinnae after exposing PV to various concentrations of AsV.

### Uptake and foliar extraction

**Figure 1:**
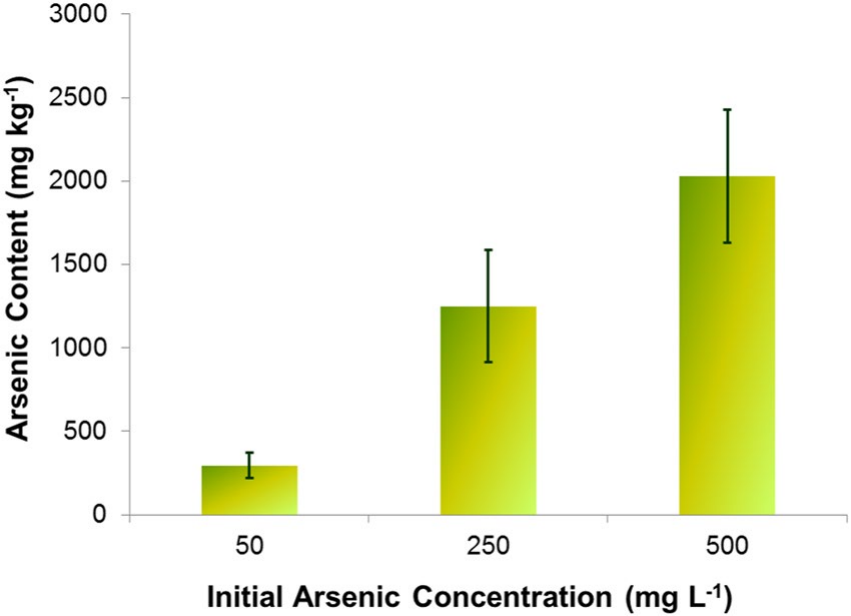
Arsenic uptake by *Pteris vittata* after 14 days. Data are expressed as mean + standard deviation.

Figure 1 shows As uptake by PV fronds as a function of initial As treatments. Plants were exposed to As solutions with initial concentrations of 0.67, 3.3 and 6.7 mM sodium arsenate for 10 days. Fronds were harvested, and used for analysis of As content, as well as subsequent extractions, SEM and XRF studies. Figure 1 shows that As content in the fronds increased linearly with increasing concentrations of As. This result supports the previously reported high affinity of PV for AsV^1^.

**Figure 2:**
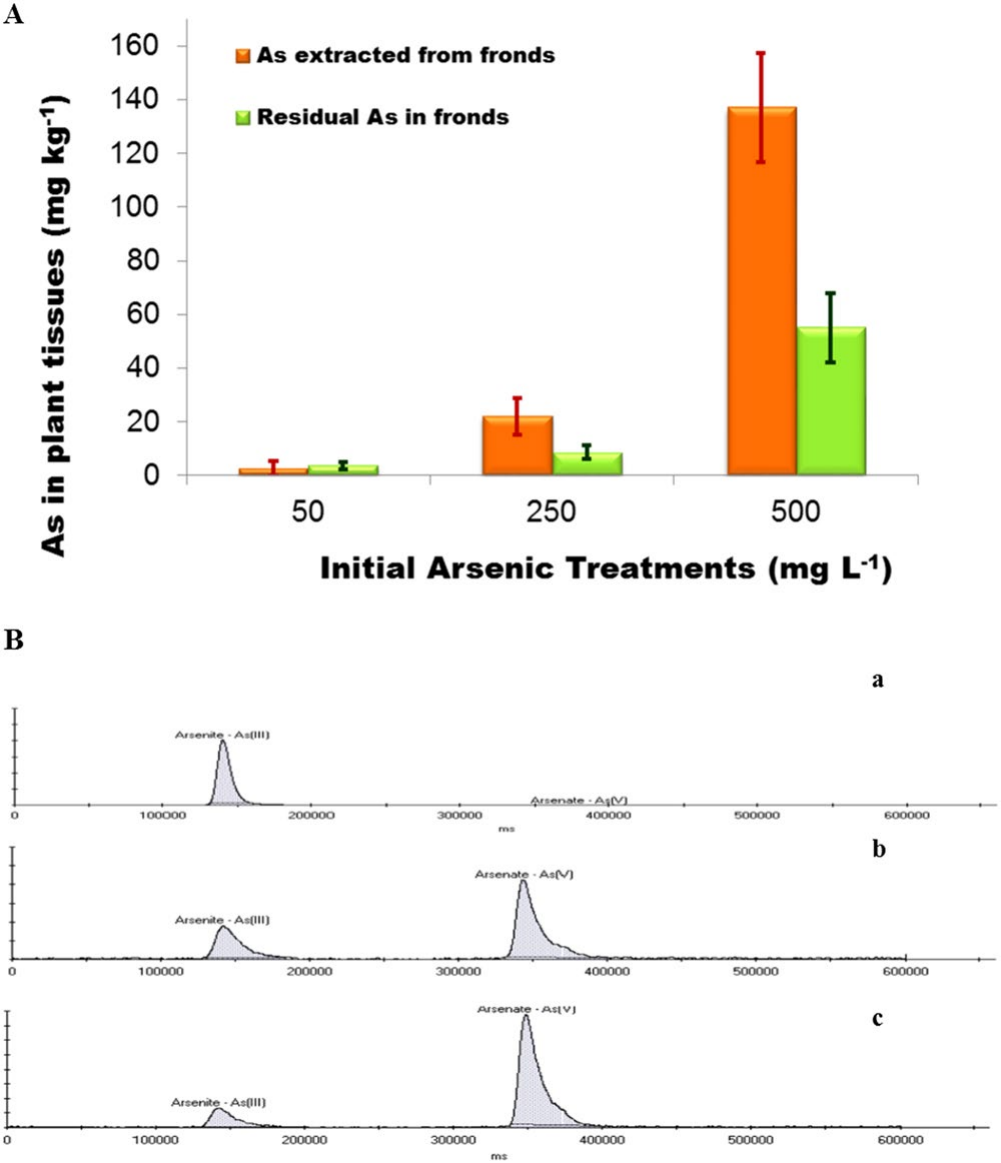
(**A**) Total arsenic extracted from the shoot using DI and total As that was retained in the shoot of *Pteris vittata*. Data are expressed as mean + standard deviation. (**B**) Speciation of arsenic in the shoot extract at a) 0.67 mM, b) 3.3 mM, and c) 6.7 mM initial As (V) concentrations. The extracts were diluted 100; 5,000 and 10,000 fold respectively for anatytical purposes.

Figure 2A shows the amount of total As (mg kg^−1^ based on wet plant weight) extracted from the fronds after being gently shaken with deionized water for 4 h. Residual As in the shoot tissues was estimated after drying and acid digestion of the fronds (Fig. 2). Fronds harvested from PV grown in 0.67, 3.3 and 6.7 mM AsV hydroponically for 14 days were shaken in deionized water for 4 h, after which they were used for As extraction to evaluate the remaining As in the tissue. The results showed significant (p < 0.001) removal of As; 43%, 72%, and 71% of total As were extracted from fronds treated with 0.67, 3.3 and 6.7 mM As respectively. A recent study reported that when PV was grown in sterile hydroponic media containing 37.5 mg kg^−1^ AsV, 95% of the As in the media turned to AsIII after two months, suggesting efflux of AsIII by the roots following uptake^22^. Similarly, in rice treated with AsV, AsIII is extruded, facilitated by an membrane transport protein in the root tips^23^. In shoot tissue, a loss of about 2–7% of As through foliar leaching was reported^25^, but our extraction results indicate much higher foliar loss, in a concentration dependent manner (Fig. 2A). Figure 2B shows the speciation of As extracted from the fronds. In fronds treated with 0.67 mM As(V), only AsIII was detected, but in fronds treated with 3.3 and 6.7 mM of As(V), both AsIII and AsV were detected.

### SEM-EDX Analysis

**Figure 3:**
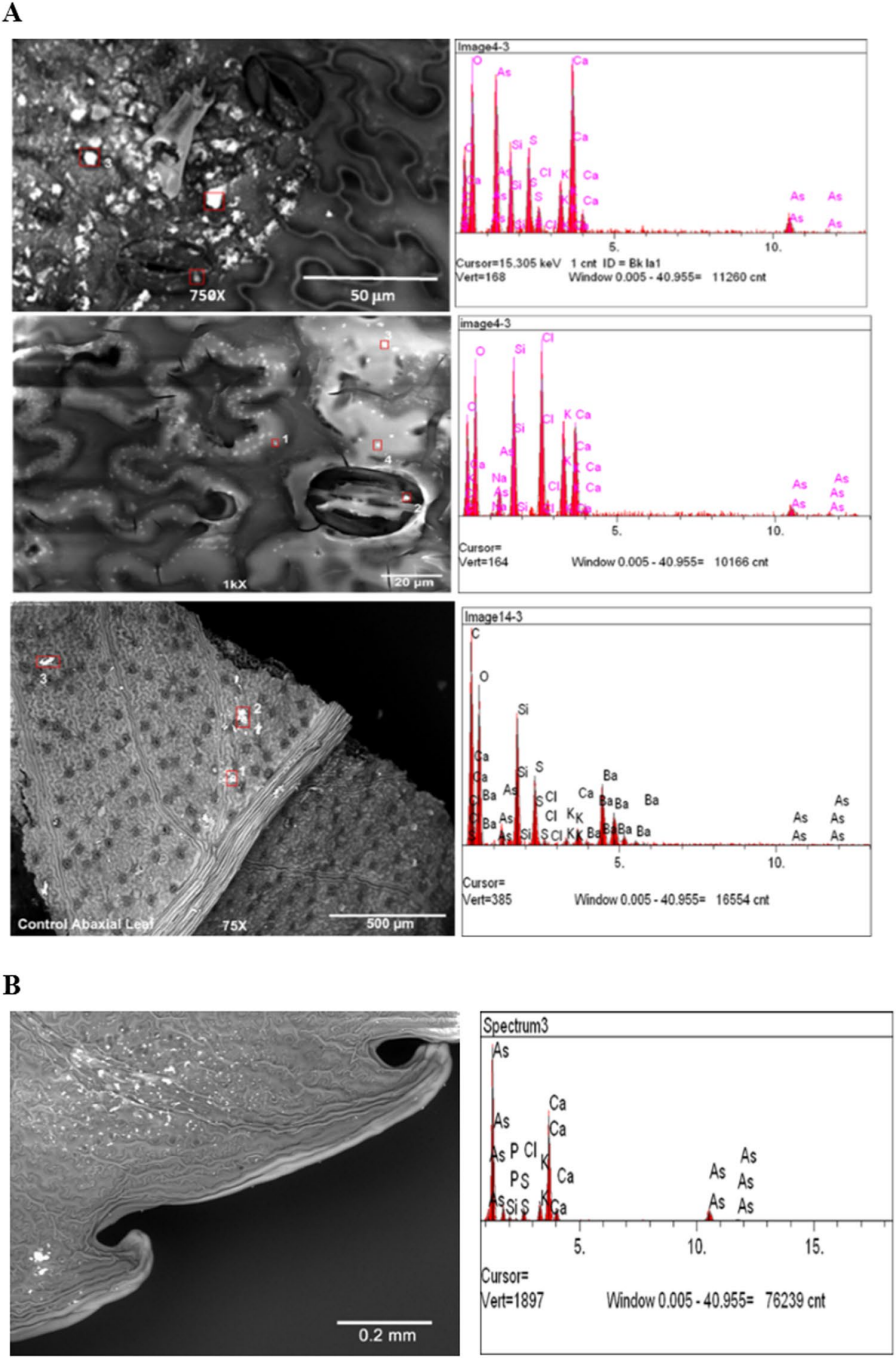
(**A**) SEM-EDX mapping of the abaxial surface of As (V) treated leaflet treated with 13.3 mM AsV. SEM images of PV pinnae (left) showing crystalline deposits. Multiple spots on each sample (marked by red squares) were analyzed by EDX (right). Top Panel: Arsenic-treated pinna before extraction, Middle Panel: As-treated pinna after extraction, Lower Panel: Control pinna (untreated). (**B**) SEM-EDX mapping of the adaxial surface of As (V) treated leaflet treated with 13.3 mM AsV. Spots on were analyzed by EDX (right).

**Figure 4:**
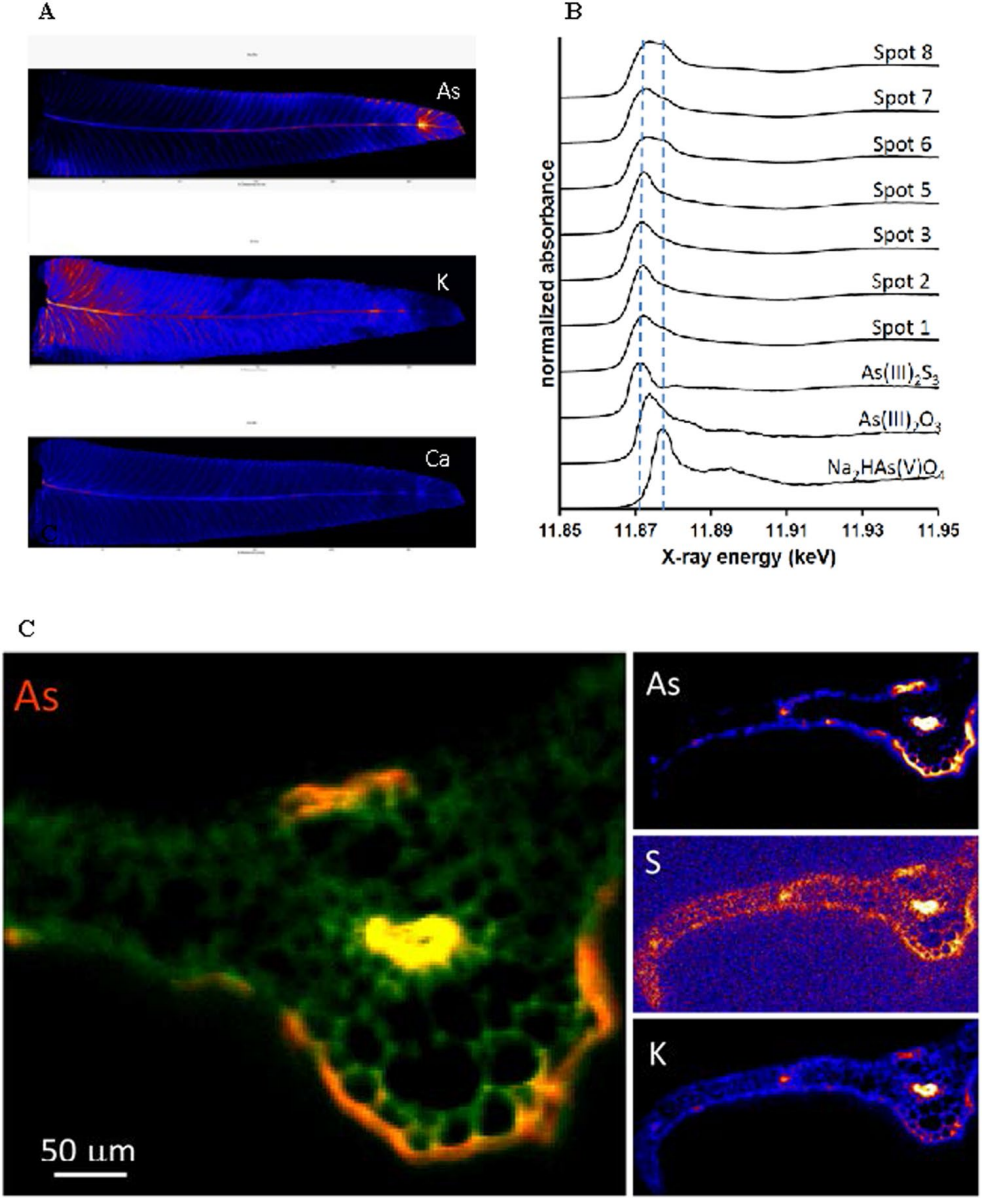
(**A**) Synchrotron analysis of a leaflet initially treated with 6.7 mM AsV showing the distributions of As as compared to K and Ca. (**B**) Arsenic K-edge µ-XANES scans collected at various locations throughout the leaflet shown in 4 A (As), compared to AsV and AsIII reference spectra. The two dashed lines locate the energy positions of the edge maxima of As(III)2S3 and Na2HAs(V)O4. The location of the spots are detailed in Supplementary Information Fig. 1. (**C**) Synchrotron X-ray fluorescence images of As, S and K in pinna tissue cross-section (2 mm from apex) from 6.7 mM treatment showing elevated levels of As adjacent to mid-rib vein and secondary veins with preferential accumulation on abaxial (lower) surface of the pinnae.

Figure 3 shows the SEM-EDX mapping of As at the abaxial (lower) (A) and adaxial (upper) (B) surfaces of a PV leaflet, treated with 13.3 mM AsV. Treated fronds were frozen in liquid nitrogen, freeze fractured and freeze-dried for 24 h. Fronds grown in As were observed by SEM before and after the extraction experiment; plants grown without As were used as control. Arsenic deposits were mainly located at the abaxial surface (Fig. 3 – top panel), which corroborates the X-ray fluorescence imaging results (Fig. 4). Some As deposition was also found on the adaxial surface (Fig. 3 - middle panel), while control pinnae showed no As deposits (Fig. 3 – bottom panel). This result is similar to that of Cantamessa *et al*.^25^, which indicated that As leaches out through hydathodes. The abaxial surface had larger number of As deposits, located sporadically throughout the lower surface of the leaf, with larger clusters appearing more frequently near stomata (Fig. 3 – middle panel), hence As could be removed through both hydathode and stomatal openings. However, further experiments are needed to verify this result. X-ray spectrum of the deposits showed that As was present together with Ca (and probably O), although smaller amounts of other elements (P, K, Cl, S) were also present. Similar X-ray spectra were obtained for the deposits on the adaxial surface. Similar results were obtained at 10 mM As treatment, but As could not be visualized in the cells, cell walls or surface by SEM at lower concentrations of 0.67 and 3.3 mM As (data not shown).

### Synchrotron Analysis

Figure 4A shows the distribution of As, K and Ca in a PV leaflet treated with 6.7 mM As. Arsenic accumulated in the veins and along the margins of the pinnae (Fig. 4A). The pinna tip was particularly enriched in As, whereas the base and the tissues in between the veins contain less As (Fig. 4A). In contrast, K levels were higher at the base and decreased toward the tip of the pinnae, whereas Ca was mostly located at the base of the rachis (Fig. 4A). The As distribution pattern suggested that As is likely to be transported by sap flow through the veins with limited uptake into the surrounding tissues. The distribution also indicates how As is likely to be extruded from the hydathodes, which are located at the vein tips.

XANES scans taken at locations throughout the leaflet are presented in Fig. 4B. The location of the spots used for scan are specified in Supplemental Information. The results demonstrated the presence of AsIII at all locations, with co-occurrence of AsV in some spots. Since wet tissues were used, formation of As(V) species during the single XANES scans could have occurred due radiation damage. Moreover, the XANES spectra suggest coordination of AsIII to S ligands, based on the similarity to the edge position of As(III)_2_S_3_ (Fig. 4B). The presence of thiol compounds is known to be important for As detoxification in non-accumulators, which produce As(III)-S complexes, but been generally reported to have a limited role in PV^6, 11, 26^. However, previous studies have reported a significant positive correlation between As and S^27^, and a similar distribution of low molecular weight thiols (LMWTs) and As in PV fronds^13^. Increased glutathione (GSH) and PC2 (a dimer of GSH) concentrations in PV fronds treated with As^28^ has also been reported. The presence of As(V) in the pinnae could be due to use of higher concentrations of As in the growth medium, which resulted in incomplete reduction of AsV to AsIII^18^.

### X-ray fluorescence imaging

**Figure 5:**
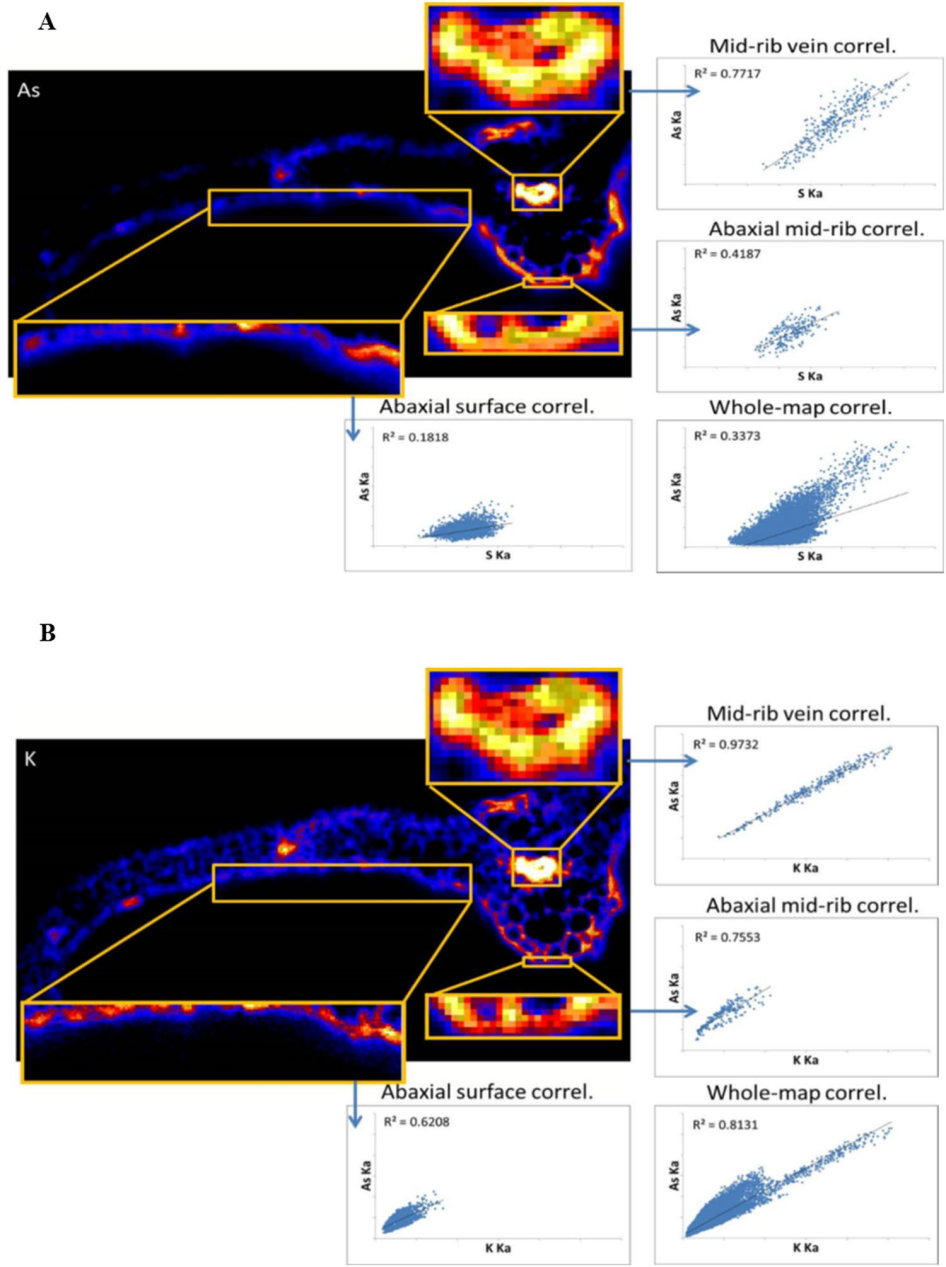
(**A**) Inset As image from Fig. 4C showing the spatial correlation of S and As in pinna and the correlations observed in highlighted regions of the mid-rib vein, abaxial surface of the midrib, and abaxial surface of the pinnae. (**B**) Inset K image from Fig. 4C showing the spatial correlation of K and As in pinna and the correlations observed in the highlighted regions of the mid-rib vein abaxial surface.

Figure 4C shows a synchrotron X-ray fluorescence image of As, Ca and K in a tissue cross-section of PV pinna treated with 10 mM AsV collected approximately 2 mm below the apex. Arsenic in the pinnae was observed to have leaked onto the pinna surface in the proximity of veins, with majority localized near the midrib (Fig. 4C). More As is observed on the abaxial (lower) surface than on the adaxial (upper) surface. Arsenic in the pinna is spatially correlated with K, Ca and S, and the correlation is strongest in the vasculature (Fig. 5A,B). Pearson correlation coefficients (R^2^) for As vs. K, As vs. Ca and As vs. S in the midrib vein are 0.97, 0.96, and 0.77 respectively. The strong positive spatial correlation between As and K in cross-section (Fig. 5A) contrasts with the apparent anti-correlation between As and K in the whole leaf maps showing depletion of K in As-rich regions including the pinna tips (Fig. 4A). A plausible explanation is accumulation of As on the pinna surface (Fig. 4C) causing shadowing of the low-energy K *K*α fluorescence signal in the whole leaf map. This apparent artifact thus provides further support for substantial As surface deposition. As shown in Fig. 5A, As-S correlation is strongest in the mid-rib vein, and is weakest near pinnae surfaces, which could be due to the transformation of As species that leaked onto the frond surface. As-thiol and AsIII species leaked onto the pinna surface are expected to partially oxidize to AsV in the ambient conditions. It could also be the result of partial oxidation of As(III) to As(V) during sample drying over the course of the XAS analysis.

It is well known that hydathodes are involved in removal of nutrients such as Na, K, Ca, P and Mg^29^, as well as heavy metals^30^ from plants. Abiotic stress including drought, heat and air pollution exacerbates foliar leaching^30^. However, there has been few detailed investigations of foliar leaching of As by PV, other than a recent study^25^ reporting secretions from hydathodes in PV pinnae containing As, indicated by SEM-EDX. The study reported 2–7% of the total As leaching out through hydathodes in the adaxial surface of the leaflets^25^ using simulated rainfall to measure foliar leaching. In this study, pinnae were gently shaken in water for 4 h, more likely to extract the leaf apoplastic fluid, which could explain the large difference the amount of As measured in the extracted liquid in this study in comparison to the earlier report^25^. More importantly, the results indicate that a majority of the As in the pinnae is contained in the leaf apoplast rather than vacuoles as previously speculated^6, 15^. Possible apoplastic distribution of As is also supported by our high-resolution imaging of pinnae cross-sections (Figs 4 and 5), which provides no indication of As compartmentalization in epidermal cells. We speculate that the extraction used in this study removed As from xylem sap and other apoplastic spaces, possibly via diffusion of exocellular As through hydathodes or the stomata. Arsenic removal was observed with increasing levels of As in the media. While color scale (Figs 4 and 5) indicates that As concentrations on the adaxial and abaxial surfaces surrounding the midrib are similar, much higher As deposition was found on the abaxial surface, compared to the adaxial surface. The As deposits were found only at higher As concentrations. Evidence using synchrotron analysis, and detailed XRF mapping of cross sections of pinnae showed that As was mainly localized at the leaf tips and veins and concentrated more toward the lower surface of the pinnae. The results raise concern that As is prone to be released back into the environment by PV after uptake, particularly at high concentrations, which might pose a significant health risk. Hence, phytoremediation using PV may not be an effective strategy in sites contaminated with high levels of As.

## Methods

### Experimental set up and Analyses

*P. vittata* plants purchased from Plant Delights Nursery, Inc., Raleigh, North Carolina. The fern were first planted in garden soil in pots and acclimated in a greenhouse for a month. The plants were then placed in a hydroponic system in half strength Hoagland’s solution and further acclimated for two weeks inside a plant growth chamber under controlled conditions (25 °C temperature and 16 h photoperiod). After two weeks, AsV stock in the form of sodium arsenate was added to achieve initial concentrations of 0, 0.67, 3.3, 6.7, 10 and 13.3 mM As. Plants were grown in triplicates, and a set of controls without As was included. Plants were harvested after 14 days and media samples were analyzed for total As using ICPMS to determine uptake by PV. In order to estimate As removal from the fronds, harvested fronds in triplicate were placed with deionized water for 4 h with gentle shaking in an orbital shaker. The wash solution was sampled at regular intervals, and analyzed for total As using ICPMS. Arsenic remianing in the frond after water extraction was estimated using ICPMS after drying the fronds followed by acid digestion. Speciation of As in the water extract from the fronds was determined using HPLC coupled with ICPMS^31^. Mean comparison was conducted using JMP version 11.

### Scanning Electron Microscopy

A JEOL 5400 LV SEM attached to KEVEX Sigma energy dispersive X-ray spectrometer (SEM-EDX) was used. Fronds from PV plants grown in 13.3 mM As before and after the extraction experiment and control fronds grown without As were subjected to SEM. All samples were viewed with at 20 kv with a working distance of 21 cm, in low vacuum mode. Fronds were freeze fractured in liquid nitrogen and dehydrated for 24 hours. Samples were mounted on carbon tape attached to aluminum stubs. For each sample, an adaxial, abaxial, and a fractured cross section was mounted and viewed.

### Synchrotron X-ray microprobe analyses

Spatially resolved micro X-ray fluorescence (µ-XRF) maps were collected in combination with micro-focused As K-edge X-ray absorption near-edge spectroscopy (µ -XANES) spectra at beamline X27 A of the National Synchrotron Light Source (NSLS) at Brookhaven National Laboratory, Upton, New York. Fresh fern pinnae from plants grown in 6.7 mM As were fixed onto slide frames with Kapton tape, and mounted on an XYZ motorized sample stage positioned at 45° incidence to the beam. A Si(111) monochromator was used for energy selection, and the beam was focused with Kirkpatrick–Baez mirrors to produce a spot size of ~10 µm × 15 µm on the sample. X-ray fluorescence was measured with a 13-element Canberra Ge array detector positioned at 90° to the incident beam. Mapping was done in fly scanning mode at an energy of 13.0 keV, using a pixel size of 0.02 mm and an integration time of 0.05 s.

Micro-XANES spectra of the As K-edge (11867 eV) were collected at various locations throughout the pinnae, using steps and counting times of 5.0 eV and 3 s, 0.5 eV and 6 s, 1.0 eV and 6 s, and 5.0 eV and 3 s in the 11767–11857, 11857–11907, 11907–11967, and 11967–12167 eV energy ranges, respectively. Two or three scans were collected per spot, and averaged to improve signal:noise. XANES spectra were also collected for the reference compounds Na_2_HAs(V)O_4_, NaAs(III)O_2_, and orpiment (As(III)_2_S_3_) to assist data interpretation of the As fern data. For all XANES scans, the monochromator was calibrated by assigning the top of the white line of the spectrum of As(V) incorporated in topaz to a value of 11875 eV. Data processing involved averaging and normalizing the XANES spectra using WinXAS 3.1^32^

### X-ray microanalysis

Freshly harvested frond pinnae grown in 6.7 mM As were oriented with the tip pointing upwards and placed into a well containing OCT (optimal cutting temperature) embedding medium (Tissue-Tek, Torrance, CA) then rapidly cooled to −20 °C for preparation of the tissue cryosections. Specimens were cryotomed at −15 °C to 20 um thick cross-sections using a cryotome CM 1900 (Leica Microsystems, GmgH., Nussloch, Germany). Sections were made at 0.5, 1 and 2 mm from the tip of the leaflets. Tissue sections were mounted onto silicon nitride windows (Silson Ltd., Bilsworth, Northampton, England) and stored in a desiccator until analysis by X-ray microprobe. X-ray microanalysis of tissue sections was conducted at the APS sector 13 GSECARS beamline using a spot size of 1 × 2 um, a pixel size of 2 um and a transit time of 100 ms.

## Electronic supplementary material

Supplementary Information
