# Supplementary material for: Evidence for exocellular Arsenic in Fronds of Pteris vittata
Source: Sci Rep. 2017 Jun 6;7:2839. doi: 10.1038/s41598-017-03194-x (PMC5460129; doi:10.1038/s41598-017-03194-x)

Evidence for exocellular Arsenic in Fronds of *Pteris vittata*

Rupali Datta[1], Padmini Das[2], Ryan Tappero[3], Pravin Punamiya[4], Evert Elzinga[5], Shivendra Sahi[6], Huan Feng[7], Jeffrey Kiiskila[1], Dibyendu Sarkar[8]*

[1]Department of Biological Sciences, Michigan Technological University, Houghton, MI 49931 USA

[2]Department of Biology, Nazareth College of Rochester, NY 14618 USA

[3]Photon Sciences Division, Brookhaven National Laboratory, Upton, NY 11973 USA

[4]Merck Research Laboratory, Merck Sharp & Dohme Corp.Rahway, NJ 07065 USA

[5]Department of Earth & Environmental Sciences, Rutgers University, Newark, NJ 07102, USA

[6]Department of Biology, Western Kentucky University, Bowling Green, KY 42101, USA

[7]Department of Earth and Environmental Studies, Montclair State University, Montclair, NJ 07043 USA

[8]Department of Civil, Environmental and Ocean Engineering, Stevens Institute of Technology, Hoboken, NJ 07030 USA

*Corresponding author: Phone: 201 216 8028; Email: dsarkar@stevens.edu

Supplementary Figure 1. Synchrotron analysis of a leaflet initially treated with 6.7 mM AsV showing the distributions of As

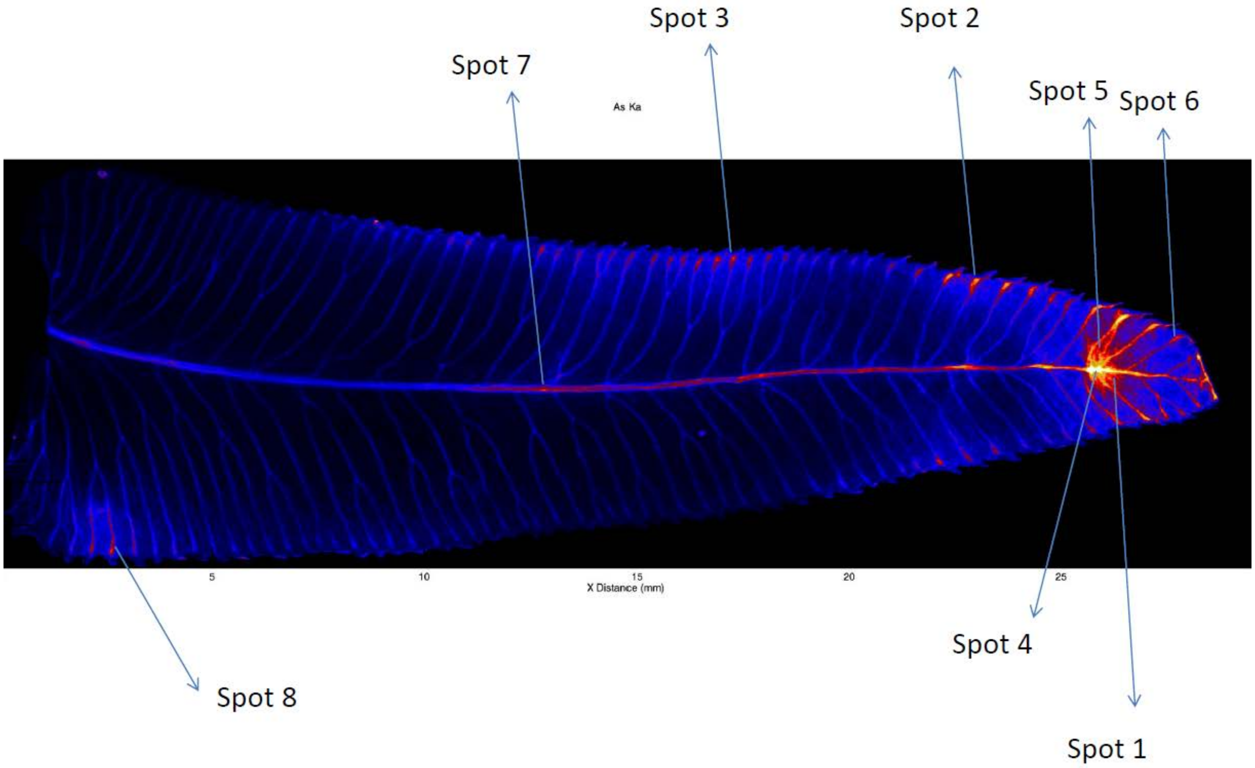

Supplemental Figure 2. Arsenic K-edge µ-XANES scans collected at various spots shown in Supplemental Figure 1. The two dashed lines locate the energy positions of the edge maxima of As(III)2S3 and Na2HAs(V)O4.

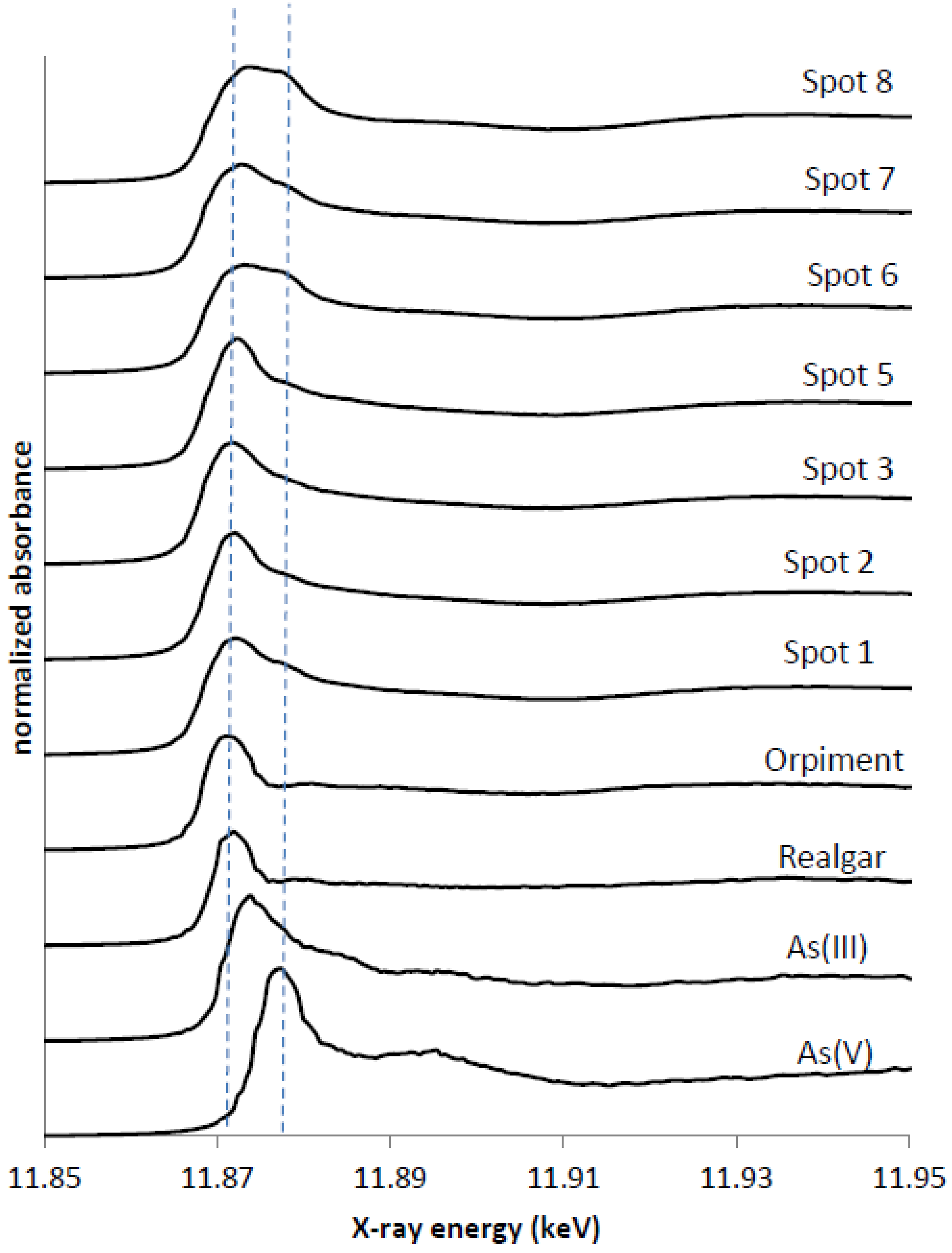

Supplement: Supplementary file 1 — Supplementary Information [file 41598_2017_3194_MOESM1_ESM.pdf]
